# Outbreak of extensively drug-resistant *Serratia marcescens* in an intensive care unit

**DOI:** 10.1017/ash.2023.454

**Published:** 2023-11-06

**Authors:** Tazio Vanni, Letícia Olivier Sudbrack, Tatiana Amabile de Campos, Rafael Nakamura da Silva, André Pitondo da Silva, Rodrigo Pereira Estefani, Tatyana Botelho de Oliveira, Paulo Henrique Caixeta Canedo, Ricardo Domingues Guzman, Jordana Rey Laureto, Julival Fagundes Ribeiro

**Affiliations:** 1 Núcleo de Controle de Infecção Hospitalar, Hospital de Base do Distrito Federal – Brasília, Distrito Federal, Brasil; 2 Organização Pan-Americana da Saúde, Departamento de Emergência em Saúde, Brasília, DF, Brasil; 3 Departamento de Biologia Celular, Universidade de Brasília – Brasília, Distrito Federal, Brasil; 4 Universidade de Ribeirão Preto, Ribeirão Preto, São Paulo, Brasil; 5 Unidade de Terapia Intensiva, Hospital de Base do Distrito Federal Secretária de Saúde do Distrito Federal – Brasília, Distrito Federal, Brasil; 6 Laboratório de Microbiologia, Hospital de Base do Distrito Federal – Brasília, Distrito Federal, Brasil

## Abstract

We present the investigation and control of an extensively drug-resistant *Serratia marcescens* outbreak in a 30-bed intensive care unit (ICU). Within 6 weeks, 4 critically ill trauma patients were infected by the same strain. Intensive containment measures limited the spread of this strain while sustaining the capacity of the trauma ICU.

## Introduction

*Serratia marcescens* observed in hospitals often presents different resistance mechanisms; however, they are rarely extensively drug-resistant (XDR).^1,2^ *S. marcescens* increasingly adapts to different hospital environments, making it difficult to identify and eliminate the source of an outbreak.^2,3^

There were reports on the containment and investigation of an outbreak of XDR *S. marcescens* in the intensive care unit (ICU) of a hospital in Brasília, capital of Brazil. We describe clinical outcomes, antimicrobial resistance, genetic relatedness of isolates, and control measures applied for outbreak containment. The study was approved by the ethics committee of IGESDF (CAAE 64402322.6.0000.8153).

## Methods

### Setting and definitions

The outbreak occurred in a 711-bed tertiary hospital with 80 ICU beds. The outbreak occurred in a 30-bed trauma/general ICU organized in a single open-plan unit with an average of 54 admissions per month. Patients admitted to this ICU are mostly polytrauma patients.

A case was defined as a positive culture result for the XDR *S. marcescens* during and after ICU admission, associated with a negative admission sample. All other patients hospitalized in the ICU were identified as contacts, by the Outbreak Control Team (OCT). The OCT was responsible for establishing and monitoring the action plan to contain and investigate the outbreak.

### Microbiological and genomic testing

Initial susceptibility testing for 13 antibiotics was performed on all isolates using MicroScan autoSCAN-4 (Beckman Coulter Diagnostics), which is currently used in the hospital laboratory.^4^ Additionally, susceptibility testing for 29 antibiotics was performed in the LABAC-UNAERP laboratory using a modified Stokes disc diffusion method on diagnostic sensitivity test agar.^5^ Genotypic tests were also carried out to investigate the main resistance genes (*bla*_KPC_, *bla*_NDM_, *bla*_VEB_, *bla*_IMP_, *bla*_VIM_, *bla*_CTX-M-Gp8_, *bla*_CTX-M-Gp9_) in the Serratia isolates.^6^

In order to assess genetic similarity among the 4 bacterial isolates, Enterobacterial Repetitive Intergenic Consensus Polymerase Chain Reaction (ERIC-PCR) DNA fingerprinting was performed.^7^ Genomic DNA from the 4 isolates was extracted using a QIAamp1 DNA Mini Kit (QIAGEN, Hilden, Germany). The primers used were: ERIC1 5'-ATGTAAGCTCCTGGGGATTCAC-3' and ERIC2 5'-AAGTAAGTGACTGGGGTGAGCG-3'. The amplified product was visualized by agarose gel electrophoresis and the band patterns were compared to each other.

## Results

### Epidemic timeline and patient characteristics

On July 26, the Microbiology laboratory notified the hospital infection control unit of a potential outbreak of *S. marcescens* resistant to all tested antibiotics, which was isolated in 3 blood cultures of different patients, within a period of 2 weeks. The 3 patients were in the Trauma ICU within 5–7 meters from each other. Despite infection control efforts for almost 3 weeks, the XDR *S. marcescens* was isolated from endotracheal tube sample of another patient. This patient was also in the Trauma ICU in close range to the initial three cases, prior to the implementation of cohort measures.

All 3 initial patients, who presented with bacteremia, had central venous lines and were under mechanical ventilation (Table 1). The index case, who had been hospitalized for approximately 2 months, was receiving hemodialysis.

**Table 1.** Patients’ characteristics, *S. marcescens* infection information, and clinical outcomes

Patient	Age (years)	Gender	Diagnosis	Day of ICU admission	Date of specimen collection	Type of specimen where the XDR *S. marcescens* was identified	Infection and risk factors associated with the XDR *S. marcescens*	Treatment	Outcome*
1	28	M	Polytrauma	22/05/22	11/07/22	Blood	Bloodstream infection. Use of CVC, MV, and HD. Subjected to decompressive craniotomy	Meropenem + Amikacin	Discharged to another health service
2	44	F	Subarachnoid hemorrhage	14/07/22	22/07/22	Blood and cerebrospinal fluid	Ventriculitis. Use of CVC, VM, and EVD. Subjected to craniotomy.	Meropenem + Amikacin	Discharged to another health service
3	58	M	Cerebral aneurysm and subarachnoid hemorrhage	16/07/22	23/07/22	Blood	VAP and sepsis. Use of CVC and VM. Subjected to craniotomy and aneurysm clipping	Meropenem + Amikacin	Discharged home
4	59	M	Empyema and Sepsis	16/08/22	24/08/22	Tracheobronchial secretions (TS)	VAP and IAA. Use of CVC and VM. CRAB is also identified in TS.	Meropenem + Tigecycline	Dead

Note.

* Outcome 30 d post *S. marcescens* diagnosis. ICU, intensive care unit; CVC, central venous catheter; MV, mechanical ventilation; HD, hemodialysis; EVD, external ventricular drain; IAA, intra-abdominal abscess; CRAB, carbapenem-resistant Acinetobacter baumannii.

As presented in Table 1, 1 patient presented with ventriculitis caused by the XDR *S. marcescens.* The combination of Ceftazidime-Avibactam and Aztreonam was considered by the infection control team. However, the 2 antibiotics were not available to treat the patients. Meropenem and Amikacin was the regimen chosen to treat most patients due to potential synergy and availability.^8^ One of the 4 patients, who was also infected with an *Acinetobacter baumannii*, was treated with Meropenem and Tigecycline.

### Microbiological and genomic results

**Figure 1.**
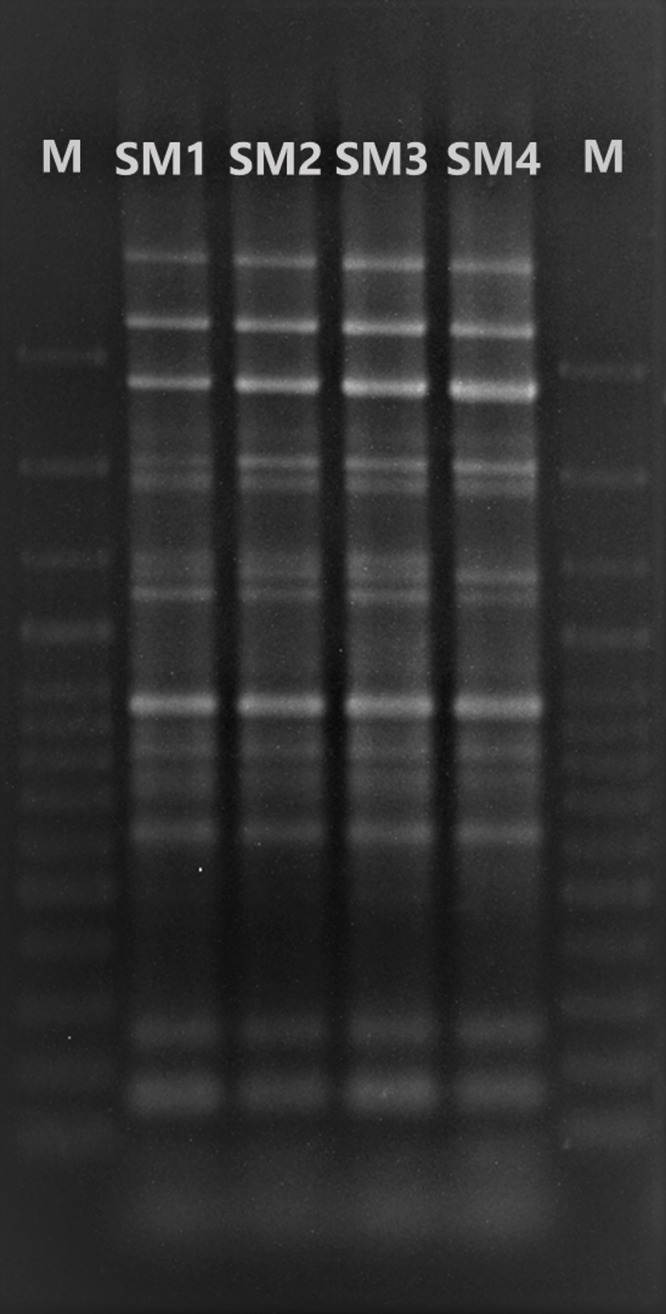
Enterobacterial Repetitive Intergenic Consensus Polymerase Chain Reaction of *Serratia marcescens* isolates from samples of 4 patients. SM1: *S. marcescens* isolate from patient 1, SM2: *S. marcescens* isolate from patient 2, SM3: *S. marcescens* isolate from patient 3, SM4: *S. marcescens* isolate from patient 4.

Initial automated susceptibility testing on the *S. marcescens* isolates from the 4 patients were resistant to all tested antibiotics. Additional susceptibility testing using a modified Stokes disc diffusion method showed that, except for Fosfomycin (intravenous formulation not available in Brazil), the isolates were resistant to all 29 antibiotics. Genomic testing of the main resistance genes in the 4 Serratia isolates detected the *bla*_NDM_ gene in all isolates, and the *bla*_KPC_ only in 2 of the 4 isolates. Strains carrying the respective genes were used as controls for PCR reactions. Nucleotide sequence accession numbers of control strains: MH818574 and MH818573. Other genes that were investigated (i.e. *bla*_VEB_, *bla*_IMP_, *bla*_VIM_, *bla*_CTX-M-Gp8_, *bla*_CTX-M-Gp9_,) were not detected. Phenotypic metallo-b-lactamases production test was positive in all 4 isolates. As shown in Figure 1, the ERIC-PCR demonstrated 100% genetic similarity among the 4 bacterial isolates and suggested horizontal transmission.

### Containment and investigation

As soon as the first patients were identified, all parties involved in the assistance, surveillance, and infection control, as well as local health authorities, were notified and control measures put in place, including a) cohorting of patients and health care professionals assisting those infected/colonized, b) creation of a closed physical border for the cohort area, c) installation of personal protective equipment stations at the entrance of the cohort area, d) reinforcement of hand hygiene and contact precaution training of health care personnel working in the trauma ICU covering all shifts, f) limitation of new admissions and patient transfer, g) weekly testing of contacts, h) strengthening of environmental and equipment decontamination protocols, i) revision of water and dialysis fluids periodic analyses, j) daily monitoring of clinical status and laboratory data of infected/colonized; and k) daily monitoring of microbiology results of the all hospitalized patients.

Investigation efforts were also put in place to identify common risk factors among the infected/colonized, as well as to review the health assistance processes, including storage, preparation, and administration of solutions and medications. The fact that the 4 patients infected with the XDR *S. marcescens* were 5–7 meters from each other, as well as were under mechanical ventilation and had central venous lines raised the possibility of patient cross-transmission.

Inspection visits to the medication and solution storage room and preparation station were performed, however, protocol deviations were not identified. During the retraining of health care personnel, we could not identify any misconception regarding preparation and administration of solutions and medications. Microbiological sampling of the environment was performed, particularly of faucets and hemodialysis water supply. However, the XDR *S. marcescens* was not identified. Periodic analyses of water and dialysis fluids were revised and results were according to standards.

Weekly testing of contacts using rectal swabs was carried out until 2 consecutive weeks without any infection or colonization were registered. All hospital microbiological test results were daily monitored for up to a month after the last XDR *S. marcescens* was detected in the hospital.

## Discussion

We report the containment and investigation of an outbreak of XDR *S. marcescens* in a trauma ICU. A single strain of XDR *S. marcescens* spread quickly in the ICU challenging the containment of this strain while sustaining the capacity of the main trauma ICU of the region. The outbreak strain was resistant to all antibiotics available at the hospital posing a serious threat to critically ill patients.

Nosocomial outbreaks of *S. marcescens* have been reported in different scenarios.^2,3^ However, there are few published reports of XDR *S. marcescens* outbreaks.^9,10^ Like other *S. marcescens* outbreaks,^2,3^ despite an extensive investigation, the source of the organism was not identified.

Finally, during the following 6 months postoutbreak, no additional infection/colonization cases of the XDR *S. marcescens* were identified. This outbreak led to improvements in the monitoring of environmental cleaning and disinfection, as well as in the active surveillance of potentially MDR-colonized patients.
